# Inductive Loop Axle Detector based on Resistance and Reactance Vehicle Magnetic Profiles

**DOI:** 10.3390/s18072376

**Published:** 2018-07-21

**Authors:** Zbigniew Marszalek, Tadeusz Zeglen, Ryszard Sroka, Janusz Gajda

**Affiliations:** Department of Measurement and Electronics, AGH University of Science and Technology, 30 Mickiewicz Avenue, 30-059 Krakow, Poland; tezet@agh.edu.pl (T.Z.); ryszard.sroka@agh.edu.pl (R.S.); jgajda@agh.edu.pl (J.G.)

**Keywords:** slim inductive loop sensor, vehicle magnetic signatures, impedance change, signal conditioning, axle detector, traffic measurements

## Abstract

The article presents a measurement system that captures two components of a motor vehicle’s magnetic profile, which are associated with the real and imaginary part of the impedance of a narrow inductive loop sensor. The proposed algorithm utilizes both components of the impedance magnetic profile to detect vehicle axles, including lifted axles. Accuracies of no less than 71.8% were achieved for vehicles travelling with a lifted axle, and no less than 98.8% for other vehicles. The axle detection accuracy was determined during a series of experiments carried out under normal traffic conditions, using profile analysis, video footage and reference signals from an axle load detector on a total of 4000 vehicles.

## 1. Introduction

Research aimed at finding new systems and methods for measuring vehicle parameters is carried out in many research centers around the world [1,2,3,4,5,6,7,8,9]. The key parameters of interest are vehicle speed, length, the presence of a trailer, front, and rear overhangs and, most importantly, the number of axles and the distance between them, which play a key role in many vehicle classification schemes [4,5]. Intelligent transportation systems (ITS) use these parameters to automatically classify vehicles, to determine traffic statistics and aggregated traffic characteristics, to verify traffic models, and for adaptive traffic control and the generation of messages for traffic participants. Traffic characteristics are constantly updated and made available from multiple locations at the same time, which makes it possible to draw the right conclusions regarding the use of road infrastructure and to support emergency services when responding to road accidents [6]. The global traffic characteristics of a given section of road infrastructure can be used to identify and predict the harmful impact of traffic on the environment and air quality. Statistics related to vehicle flow in a given area play a crucial role when developing road infrastructure such as petrol stations and parking lots. There is also a growing demand for vehicle class information in traffic control systems such as automatic entry gates, and electronic toll collection and charging systems. Automatic classification is also essential in Weigh-In-Motion systems, as the permissible axle load depends on the number and configuration of the axles of a vehicle as well as its class [7,8].

In the literature one can find a description of sensors made using various technologies and used for the detection, counting and classification of car vehicles. In addition to optical, infrared and ultrasonic sensors, inductive loops, and load sensors mounted in the road surface, various magnetic sensors, including magneto impedance [10,11], giant magneto-impedance [12,13], magneto-resistance [14], giant magneto-resistance [15], and fluxgate sensors [16] have been studied widely.

In [10] a magneto-impedance sensor used to count vehicles is described. Such sensors are mounted on the surface of a traffic lane and are more resistant to environmental effects than are other solutions using induction loops or piezoelectric cable sensors. The detector described generates a presence signal, which allows vehicles to be counted. However, the magnetic profiles presented in [10]—corresponding to different classes of vehicles—indicate this solution’s potential for being used in vehicle classification systems. Unfortunately, the authors did not present any research results making it possible to assess the accuracy of a vehicle classification system based on a magneto-impedance sensor. However, it should be emphasized that the phenomena responsible for generating a magnetic profile in an inductive loop and in integrated magnetic field sensors are completely different. In addition, the use of integrated magnetic field sensors in systems for detecting the axles of motor vehicles using a magnetic profile is still under-researched.

Systems equipped with load sensors installed in a roadway provide the most accurate measurements of the number of axles and the distance between them [9]. Although these systems are highly effective (with the exception of detecting lifted axles), they are not a low-cost solution, as they rely on relatively expensive piezoelectric sensors that have a short working life (nominal two years), as well as on quartz, tensometric, and pneumatic sensors. These detectors differ in price, operating principle and installation method. What they have in common is that their active element is exposed to the force exerted by the wheel of the passing vehicle, which is a fundamental drawback that not only limits the lifespan of the sensor but can also lead to the misclassification of vehicles with lifted axles.

The authors agree that an alternative to such expensive and unreliable (in lifted axle detection) classification systems based on load sensors may be inductive loop (IL) sensor-based systems. These offer many advantages over load sensors, including an attractive price, easy installation, independence from weather conditions, high durability, and the ability to detect lifted axles [17]. In addition, IL sensors are now the second most common source of traffic information after traffic cameras. If slightly modified, they have the potential to significantly increase the amount of information obtained about a vehicle by providing data on the number and location of its axles.

Currently, IL sensor-based systems for vehicle axle detection are not commonly used in ITS due to their lower detection rate when compared to load sensors. Sources claim that various research centers have been, and still are, developing this technology [17,18,19], focusing primarily on increasing the accuracy of axle detection [20]. The results of some of these projects have already been patented [21,22,23,24].

The idea of using an IL for axle detection was first discussed in [21]. In that solution, the IL is rectangular and its dimension in the direction of vehicle movement is smaller than the wheel diameter. The IL is part of the resonant circuit of an LC generator. The output signal of the generator undergoes frequency demodulation (FM demodulation). The signal generated at the output of the demodulator—known as a magnetic profile—indicates the presence of those metal parts of the vehicle with the lowest geometrical location, primarily the steel belt of a tire (a ferromagnetic steel belt reinforcement commonly used in tubeless tires). Despite the special design of the narrow IL sensor, the magnetic profile also registers artifacts generated by the chassis and its low-suspended elements, which limits the effectiveness of axle detection, particularly in passenger vehicles with low suspension. The accuracy of this solution in traffic conditions has not been determined.

To increase the accuracy of axle detection, Robert Harper Lees [22] used an IL sensor that, in terms of its shape and the layout of the wire in the pavement, resembled the number 8. This patented solution [22,23] is known as an Idris loop. The dimension of the Idris loop along the lane is not greater than 60 cm and the electromagnetic field it generates does not exceed 30 cm above the road surface. In order to increase the accuracy of automatic vehicle classification (AVC) systems, a set of octal and rectangular IL sensors is used. A dedicated algorithm analyzes the magnetic profiles obtained from these sensors and extracts information to classify the passing vehicles. A complete AVC system is currently offered by Peek Traffic [24] under the name Peek Traffic Idris SmartLoop AVC.

Modifications in the shape and orientation of the IL sensor along the lane in order to measure additional parameters related to the vehicle led the authors of [19] to construct a very narrow sensor, known as a “Blade”, which is characterized by its diagonal alignment with respect to the lane. The “Blade” sensor works with a conditioning system equipped with two LRC generators. One generator is connected to the sensor, and the second functions as a reference system. Both generators are simultaneously energized by an electrical impulse. The resulting differential signal is converted into digital form and recorded. The signal obtained carries information about the chassis and wheels of the vehicle. These signals can be further processed to determine vehicle presence and class.

The research article [17] shows an application in which a rectangular, narrow IL sensor works with a Maxwell–Wien bridge. The bridge is pre-balanced in the absence of a vehicle. The output voltage of the bridge undergoes synchronic demodulation. The phase of the sync demodulator signal was adjusted experimentally such that the impulses generated by the wheels of a vehicle belonging to the class in question were visible in the output signal. The axle detection accuracy of an IL bridge-based system was compared with the piezoelectric load sensor system; the accuracy was established by comparing the number of vehicles in which the number of axles was correctly counted with the total number of vehicles in a given class. The best results were achieved for 5 and 4-axle vehicles (99% and 96%, respectively). The results were worse for vehicles with steel rims (95%) and for 3-axle vehicles (92%). The axles of vehicles with aluminum rims were detected with an accuracy of over 88%. In this solution, axle detection accuracy was affected by the setting of the phase angle of the sync demodulator signal. The setting that allowed a high rate of detection of wheels with aluminum rims was different than that for steel rims, which is considered a disadvantage of this solution. All things considered, the results have shown that narrow IL sensors can be used for axle detection and for determining the number of axles.

In summary, there are currently many technologies available for detecting vehicles and determining their characteristic parameters. Those most commonly used include hydraulic, pneumatic, reluctance, resistive, optical fiber, capacitive, piezoelectric, quartz, tensometric, inductive loop, magnetic, acoustic, ultrasonic, laser, microwave and infrared sensors, light curtains, and cameras with image processing algorithms. When it is necessary to detect the axles of vehicles and to measure their distribution in the body of a vehicle (e.g., in WIM systems), this group in principle narrows down to load sensors. An alternative is inductive loop sensors of a specific construction [19,21,22,24], which have the advantage of being simple, durable and relatively inexpensive to install and maintain, although high detection efficiency is a problem. However, the authors of these publications do not analyze the possibility of detecting lifted axles, which is extremely important in order to classify vehicles correctly, especially heavy goods vehicles. This problem was addressed in work [17], which pointed out a number of problems related to the construction of rims (steel or aluminum) and the effective detection of axles. The present paper refers to [17], using the same sensor construction, but proposes a different conditioning system and a data processing algorithm to solve the problems identified in [17].

## 2. Inductive Loop Vehicle Detector—Principle of Operation

The principle of operation of an IL sensor is that the metal components of a vehicle affect the variable electromagnetic field produced by the IL, powered by sinusoidal alternating current, which in consequence changes the observable parameters of the sensor impedance. These changes in parameters cause various effects, depending on the electronic circuit cooperating with the IL sensor. At the output of the generator systems there is a change in the resonant frequency [25]; in AC-bridge systems there is a change in output voltage; in circuits with a phase-locked loop there is a change in voltage that determines the frequency of the voltage-controlled oscillator [26]. Changes in these values, represented as a function of the time or distance travelled by the vehicle passing over the IL, are called the magnetic profile of the vehicle. In many applications, the magnetic profile is essential for vehicle detection and classification [4,5,18].

Signal changes in the magnetic profile are mainly influenced by two phenomena that occur during the presence of the vehicle in the sensor field, i.e., the flow of eddy currents and the ferromagnetic effect [27]. In the impedance model of the IL sensor (*Z* = *R* + *j*X), the phenomenon of eddy currents reduces the reactance value in this model (*X*) and increases the resistance value (*R*).

The ferromagnetic effect enhances the magnetic flux associated with the IL (in the presence of ferromagnetic elements such as the steel belt of a tire) and increases the inductance (or reactance) of the IL sensor. Changes in the impedance parameters of the IL sensor are processed to the magnetic profile signal in a conditioning system [28]. This principle of operation is very similar to that in eddy current sensors for measuring displacement or thickness, and has been used also in proximity detectors and in the nondestructive testing of metallic objects [13,15].

## 3. Inductive Loop Axle Detector

In contrast to standard vehicle detectors, the IL axle detector proposed in this paper consists of three main elements:

(1) A narrow, rectangular IL sensor, with a length in the direction of vehicle movement of 10 cm and a width of 3.2 m, comprised of 4 turns of wire, with nominal impedance parameters of *Z* = 1 + *jω*(80 × 10^−6^) Ω, where *ω* is the pulsation of the alternating current of the sensor excitation;

(2) An electronic circuit cooperating with the narrow IL sensor, generating two output signals proportional to the changes in the real and imaginary part of the sensor impedance, described in detail in the next section. These signals are called the resistance magnetic profile (R-profile) and the reactance magnetic profile (X-profile) of the vehicle;

(3) An algorithm for combining the information from the profiles and performing the axle detection.

### 3.1. Method for Measuring the R-Profile and X-Profile of a Vehicle

The disadvantage of resonance and AC-bridge systems is that the output voltage of the system is a nonlinear function of the change in IL sensor impedance, while the disadvantage of the LC-generator is that the signal is proportional only to the inductance, i.e., the signal that is proportional to the changes in the real part of the IL sensor impedance is lost [28]. The nonlinearity of AC-bridges forces them to work under a small imbalance, and this significantly limits the sensitivity of the method within an acceptable linearity error.

**Figure 1:**
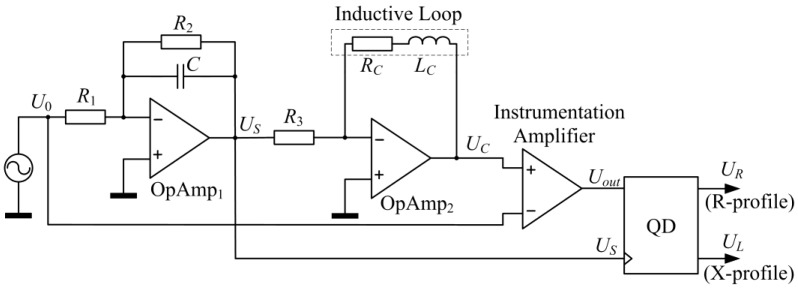
Sensor amplifier, where QD—Quadrature Demodulator (details shown in Figure 2).

The solution proposed here is free from the above disadvantages. The IL co-operating system allows linear transformations of the sensor impedance components into voltage signals (magnetic profiles) and ensures high sensitivity. A diagram of the conditioning system cooperating with an IL sensor (sensor amplifier) is shown in Figure 1.

The input signal $U_{0}$ of the system is a sinusoidal signal with a constant amplitude and frequency. The voltage $U_{S}$ at the output of the first stage can be described by the Equation (1).

(1) $$U_{S}=-U_{0}\cdot \frac{R_{2}}{R_{1}\left(1+j\omega R_{2}C\right)}$$

The $U_{C}$ voltage at the output of the second stage is equal to:

(2) $$U_{C}=-U_{S}\cdot \frac{R_{C}+j\omega L_{C}}{R_{3}}$$

therefore:

(3) $$U_{C}=U_{0}\cdot \frac{R_{2}\left(R_{C}+j\omega L_{C}\right)}{R_{1}R_{3}\left(1+j\omega R_{2}C\right)}$$

With a constant instrumentation amplifier gain equal to K, $U_{out}$ output voltage is equal to:

(4) $$U_{out}=K\cdot \left(U_{C}-U_{0}\right)=K\cdot \left(U_{0}\cdot \frac{R_{2}\left(R_{C}+j\omega L_{C}\right)}{R_{1}R_{3}\left(1+j\omega R_{2}C\right)}-U_{0}\right)$$

therefore:

(5) $$U_{out}=K\cdot U_{0}\frac{\left(R_{2}R_{C}-R_{1}R_{3}\right)+j\omega \left(R_{2}L_{C}-R_{1}R_{2}R_{3}C\right)}{R_{1}R_{3}\left(1+j\omega R_{2}C\right)}$$

Taking into account relation (1), the above equation can be written as:

(6) $$U_{out}=\frac{-K}{R_{2}R_{3}}\cdot U_{S}\left[\left(R_{2}R_{C}-R_{1}R_{3}\right)+j\omega R_{2}\left(L_{C}-R_{1}R_{3}C\right)\right]$$

If the following equations are fulfilled: $R_{2}R_{C}-R_{1}R_{3}=0$ and $L_{C}-R_{1}R_{3}C=0$, then the output voltage of the amplifier $U_{out}=0$, and the change in this voltage will show changes in the impedance components due to the presence of a vehicle in the IL sensor field. It is therefore necessary to pre-balance the sensor amplifier (no metal objects within the operating field) by appropriately selecting the resistance values $R_{2}$ and $R_{3}$, or $R_{2}$ and $R_{1}$.

The change in sensor impedance components (real and imaginary parts) caused by the presence of a vehicle throws the system out of balance. The change in output voltage $\Delta U_{out}$ can be described by the following equation:

(7) $$\Delta U_{out}=\frac{-K}{R_{2}R_{3}}\cdot U_{S}\left[\left(R_{2}\left(R_{C}\pm \Delta R_{C}\right)-R_{1}R_{3}\right)+j\omega R_{2}\left(\left(L_{C}\pm \Delta L_{C}\right)-R_{1}R_{3}C\right)\right]$$

thus:

(8) $$\Delta U_{out}=\frac{-K}{R_{2}R_{3}}\cdot U_{S}\left(R_{2}\cdot \Delta R_{C}+j\omega R_{2}\cdot \Delta L_{C}\right)$$

and:

(9) $$\Delta U_{out}=\frac{-K}{R_{3}}\cdot U_{S}\left(\Delta R_{C}+j\omega \cdot \Delta L_{C}\right)$$

Assuming that $U_{S}$ is a reference signal, then in accordance with (9), the change in the IL sensor impedance components $\Delta R_{C}$ and $\Delta L_{C}$ will independently affect the changes in the real and imaginary parts of the conditioning system output signal.

It is thus possible to independently monitor the changes in these parameters, provided that two synchronous demodulation channels are used, controlled with a $U_{S}$ signal, and a signal shifted 90° with respect to $U_{S}$.

**Figure 2:**
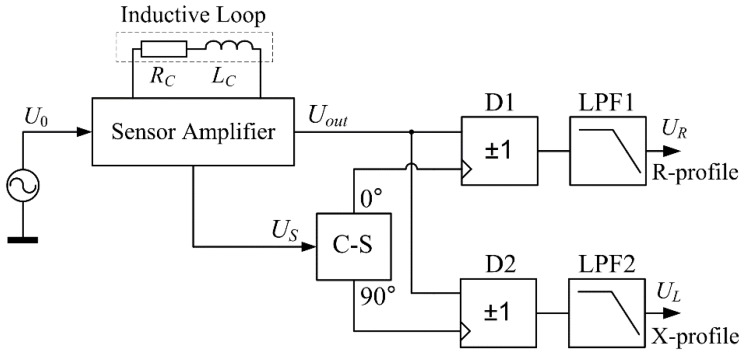
Block diagram of the conditioning system cooperating with the IL sensor comprised of a sensor amplifier (Figure 1), where: the C-S block provides two signals shifted by 90 degrees in relation to each other; D1, D2—synchronous demodulators; LPF1, LPF2—low-pass filters.

A block diagram of the overall conditioning system cooperating with the IL sensor is shown in Figure 2.

The output signal of the prebalanced sensor amplifier is passed on to two channels of sync demodulators, wherein the synchronization signals for demodulators D1 and D2 are formed on the basis of the $U_{S}$ voltage. They are obtained in the C-S block using a phase-locked loop (PLL) system, which is synchronized with the $U_{S}$ signal. The PLL works with a synchronous frequency divider composed of two JK–MS flip-flops. In the PLL synchronization state, two rectangular signals are generated at the outputs of the flip-flops. The first is synchronized with the $U_{S}$ signal, while the second is shifted by exactly 90°. These signals synchronize the demodulators D1 and D2 as well as the demodulation channels of the real and imaginary parts of the $U_{out}$ signal. The output voltages of the demodulators after low-pass filtration (in the LPF1 and LPF2 filters) are thus proportional to the changes in the impedance components of the IL sensor.

**Figure 3:**
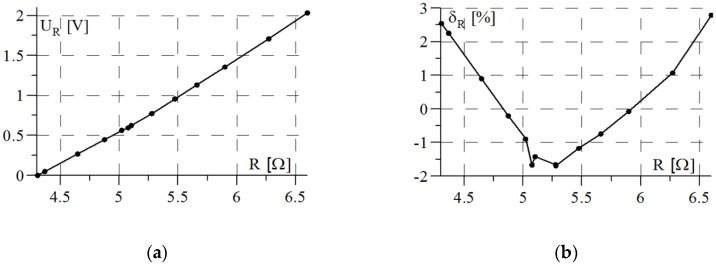
Characteristics of inductive sensor resistance measurement channel: (**a**) static; (**b**) relative nonlinearity error.

**Figure 4:**
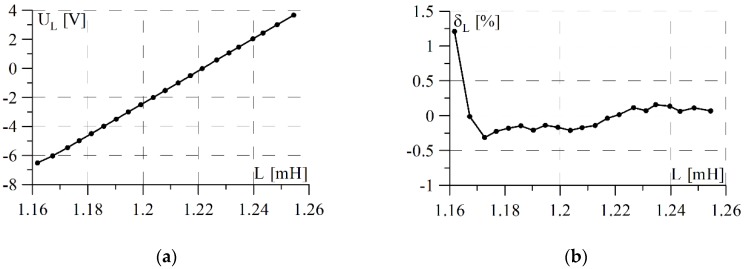
Characteristics of inductive sensor inductance measurement channel: (**a**) static; (**b**) relative nonlinearity error.

A prototype of the measurement system was subjected to laboratory tests using a small-scale inductive sensor. The purpose of the study was to determine the static characteristics of the system and identify any processing errors. In the course of the study, metal objects with different magnetic properties were introduced into the sensor operating field and the output voltage of the system was measured each time. For each case, the sensor impendence parameters were measured by means of an Agilent E4980A Precision LCR Meter. The results obtained are presented in Figure 3 and Figure 4 in the form of static characteristics and their nonlinearities with respect to linear regression.

The sensitivity of processing the inductance changes of the sensor to voltage ($U_{L}$) is many times greater than the sensitivity of its resistance changes, while at the same time the linearity of the inductance change characteristic is improved.

### 3.2. Methodology

**Figure 5:**
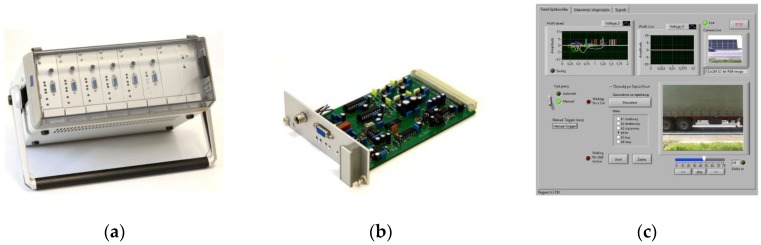

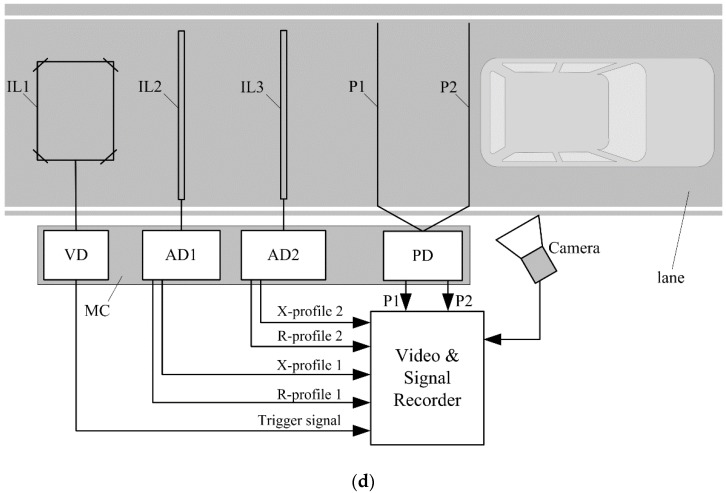
Multichannel measurement system: (**a**) multichannel conditioning system; (**b**) single card of the system; (**c**) graphical user interface of the system; (**d**) research stand diagram, where: IL1—sensor for vehicle detection; IL2 and IL3—narrow IL sensors for axle detection; P1 and P2—piezoelectric load sensors for axle detection; MC—multichannel conditioning system (shown in Figure 5a) equipped with dedicated cards for individual sensors (VD—vehicle detector card; AD1 and AD2—axle detection cards, PD—card for piezoelectric sensors).

Based on the previously formed conclusions, a multichannel measurement system was constructed, as shown in Figure 5. A single card for the R-profile and X-profile measurement is shown in Figure 5b. Video and signals recorder software (Figure 5c) was designed to simultaneously record the output voltage of the systems providing the magnetic profiles, signals from piezoelectric sensors (10 kHz sample rate)—used as reference axle detection system—and video footage (a 2-s video with a resolution of 240 × 320 px, AVI format, and 30 fps). The multichannel measurement system was used on a road stand (National Road 81, Gardawice, Poland), as shown in Figure 5d. 

The first (IL1) sensor is used to detect vehicle presence and trigger the recording of signals coming from subsequent system cards (AD1, AD2, PD and the camera). The output signals from the piezoelectric axle detectors (P1, P2) provide reference data needed to test the accuracy of the IL axle detector system, which is defined as:

(10) $$E_{\det}=\frac{L_{k}}{N}\cdot 100\%$$

where: $L_{k}$—determines the number of *k*-axles of vehicles in which all axles are correctly detected, i.e., according to the reference data, *N*—the number of vehicles in the tested group.

Vehicles were arbitrarily divided into groups according to Table 1.

Dividing was performed offline, based on the recorded data and video footage. Vehicles equipped with the same number of axles and having similar suspension were included in the same group.

**Figure 6:**
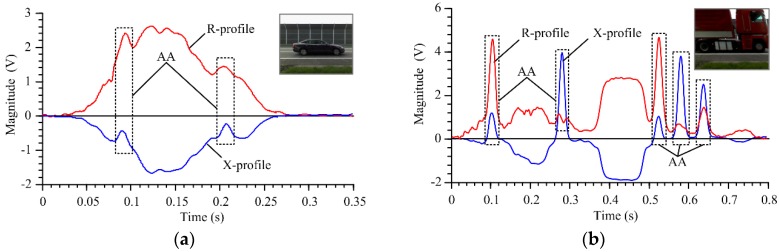
Examples of R- and X-profiles from narrow IL sensor: (**a**) passenger vehicle (low suspension); (**b**) truck vehicle (high suspension); where: AA—refers to the areas where there are artifacts originating from the vehicle axles.

Examples of a typical R-profile and X-profile from the narrow IL sensor for a passenger vehicle (passenger car with steel rims: group 2 in Table 1) are shown in Figure 6a. Sample profiles for a truck with a characteristics box located between axles 2 and 3 (group 7 in Table 1) are shown in Figure 6b.

The undercarriage of a passenger vehicle, especially the floor, consists of elements made of metal flat sheeting. When these elements are in the alternating magnetic field of IL sensor, they act as a compact coil in which eddy currents are induced. The eddy currents generate a secondary magnetic field in the opposite direction (Lenz’s law) and cause additional ohmic losses in the metal element embraced by the IL sensor field. The imaginary part of the IL sensor impedance, the inductive reactance, is proportional to the resultant magnetic field strictly expressed as magnetic flux. The real part of the IL sensor impedance, the resistance, is proportional to the ohmic losses. For these reasons, eddy currents decrease the X-profile value and ohmic losses increase the R-profile value. However, the metal elements nearest to the narrow IL sensor are the steel belts of the tires. A steel belt is a ferromagnetic element that acts as a ferromagnetic core and whose presence increases the reactance of the narrow IL sensor. For this reason, in the X-profile of a passing vehicle, local maximums are visible, marked as AA in Figure 6. It should be noted that the eddy currents induced in the flat elements of the vehicle undercarriage are a dominant phenomenon, especially in the case of passenger vehicles (Figure 6a). In truck vehicles (Figure 6b), the steel belts and tires are large, and the suspension is high, which makes the artifacts originating from the axles more visible.

### 3.3. Vehicle Axle Detection Algorithm-Core

**Figure 7:**
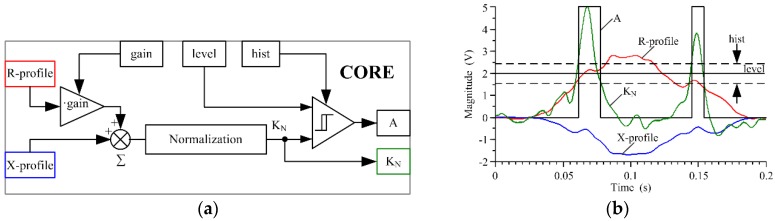
Algorithm-core of vehicle axle detection: (**a**) functional diagram; (**b**) example illustrating the principle of operation and signal processing, where: R and X-profile are input signals, gain, level, and hist are the setting parameters, A and K_N_ are the output signals.

A functional block diagram of the proposed axle detection algorithm-core is shown in Figure 7a. The R-profile obtained from the conditioning system is enhanced in the gain block, and then added to the X-profile. The gain value is properly selected for a given vehicle group so as to highlight the artifacts coming from the vehicle axles. The resulting signal samples are divided by the maximum value found before in this signal, and the obtained signal samples are then multiplied by 5; in this way a K_N_ signal is computed, which is available at the output of the normalization block. The normalized signal is then transferred to the input of the programmable comparator with hysteresis, where: ‘level’ refers to the reference level of the comparator, and ‘hist’ refers to the width of the hysteresis loop. Sample results of the detection algorithm-core execution, along with input signals: R-profile and X-profile, and output signals: A and K_N_, are shown in Figure 7b.

Preliminary research has shown that the accuracy of axle detection depends on the height of the vehicle suspension, and this in turn on the number of axles. The parameters of the axle detection algorithm-core, which are ‘gain’, ‘level’ and ‘hist’, were optimized with respect to the criterion (10) for each of the considered vehicle groups. The optimal values of these parameters are summarized in Table 2.

## 4. Discussion 

The R- and X-profiles registered are the combined effect of all of the metal components of a vehicle, including wheels and undercarriage. From the point of view of correct axle detection, the undercarriage has an impact as disturbances, whose intensity is greater the closer the undercarriage is to the IL sensor. Therefore, in vehicles with low suspension (passenger cars), the correct detection of axles is much more difficult and requires the selection of specific values for the algorithm-core parameters.

The difference in the optimum axle detection algorithm-core parameters for each vehicle type indicates a need to make an initial distinction between all vehicles as either low or high suspension vehicles (Table 2). A heuristic parameter was used for this purpose:

(11) $$D=\frac{L_{x}}{L_{m}}\cdot 100\%$$

where: $L_{m}$—number of all samples of X-profile, Lx—number of positive sample values in X-profile.

Compliance with the condition that *D* > 10% indicates that the vehicle has high suspension. Otherwise, it is assumed that the suspension is low. As the result of the research, an optimal setting of algorithm-core parameters was adopted for high-suspension vehicles: gain = 0.21, level = 0.8, hist = 0.45, and for low-suspension vehicles: gain = 0.5; level = 1.8; hist = 0.5. Vehicle axle detection accuracy (10), using an algorithm-core with an optimal setting (Figure 7a), is presented in Table 3 [20].

An analysis of the results presented in Table 3 indicates a need to modify and expand the algorithm-core to reduce the number of problems with:

(1) Incorrect detection of one axle in 2-axle vehicles (see Table 3, red value in column 1). This problem does not occur for vehicles with more than two axles.
(2) Incorrect detection of more than two axles in 2-axle vehicles (see Table 3, columns 3–5).
(3) Low detection accuracy of a lifted axle in trucks, only 2.2%.

**Figure 8:**
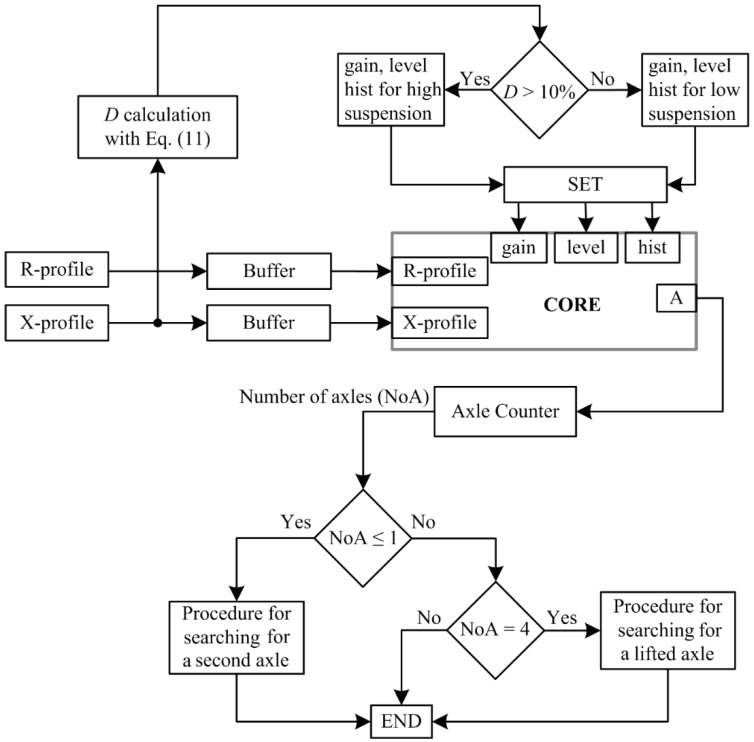
Vehicle axle detection algorithm based on R- and X-profiles.

In consequence, a novel algorithm as shown in Figure 8 was proposed. An additional procedure for detecting a second axle is triggered upon the detection of a single-axle vehicle. In this procedure, the reference level of the comparator is gradually lowered until the second axle of the vehicle is detected or the level reaches a minimum value.

The detection of excessive axles in 2-axle vehicles is caused by low-hung ferromagnetic elements (bumpers, rockers, pipes, springs, rods, etc.). These generate high-amplitude pulses in the K_N_ signal (Figure 7b), which is generally lower than in the case of pulses from wheel elements. The procedure introduced for determining the presence of a second axle and the initial analysis of the height of the vehicle suspension in the case of low-suspension vehicles makes it possible to increase the value of the ‘level’ parameter from its initial value of ‘level = 1.8′ to a much higher value of ‘level = 4′ at the initial analysis stage. 

It was assumed that the K_N_ signal (in the algorithm-core, Figure 7a) is normalized to 5, i.e., 100%. The normalized signal is then transferred to the input of the programmable comparator. Level 4 can be defined as 80%, while level 1.8 is equal to 36% of the nominal range of 5.

In comparison with impulses from wheels rolling on a road surface, impulses occurring in the K_N_ signal from a lifted axle have a much lower amplitude. It has been shown that a high lifted axle detection accuracy is achieved for level = 0.4, hist = 0.02 and gain = 0.68 (see Table 2). This means that, in order to detect lifted axles of a truck, the algorithm-core should be started with a new setting. The principle of operation for the procedure for determining the presence of a lifted axle is as follows: if four axles are detected, the result is saved. The algorithm-core is then restarted, but with settings adjusted for the detection of a lifted axle. If at the first step they do not result in the detection of a lifted axle, then a procedure is started that gradually lowers the reference level of the comparator until either a lifted axle is detected or the level reaches the minimum value (level = 0.1). If an axle is detected, the system verifies whether it is located between the second and fourth axle.

Table 4 shows the accuracy of vehicle axle detection for vehicles belonging to the 8 groups considered, according to the proposed algorithm, supplemented by the procedure for detecting a second axle and a lifted axle.

A lifted axle was correctly detected in 71.8% of trucks. In addition, the procedure for searching for a lifted axle in the profiles of 4-axle trucks was tested. There were no faults found with the procedure. However, it must be emphasized that, in the case of the 4-axle trucks, the impulse values in the R and X profiles obtained from the narrow IL sensor were relatively high. 

Comparing Table 2 with Table 3, where the algorithm was supplemented by the procedure for searching for a second axle, and the level was changed from 1.8 to 4 (Table 3), the significant difference in accuracy is because the higher level reduces the adverse effects that come from the low-suspended ferromagnetic elements of the vehicle (bumper, rocker, pipe, springs, rods, etc.). These elements have shown up as disturbances in the K_N_ signal.

The multichannel measurement system, whose structure is shown in Figure 5d, was used on a road stand, where the experimental research was carried out. The measuring stand is located in Poland on national road No. 81. It is a two-lane road, and the system is installed in the slower lane. At the place of measurement the speed limit is 70 km/h. Traffic density in this location is around 10,000 vehicles per day, and the flow of vehicles is free (no stops or real speed changes). The traffic structure is mixed (around 70% passenger vehicles, 30% delivery vehicles and trucks). Moving the system to a location with similar traffic conditions will not affect the quality of the results. Problems could occur when locating the system in urban traffic conditions where vehicle stops and sudden changes in speed are to be expected. The authors did not conduct research under such conditions, nor does the algorithm for processing vehicle profiles take these into account.

The algorithm requires the registered the R and X-profiles to be in buffers, i.e., it can be run after the registration of vehicle profiles has been completed. The acquisition time of the profiles depends on vehicle speed, length and running path in the decision tree. This means that, in the final display, the results depend on time. If the R and X-profiles exist in memory buffers, the proposed algorithm is performed on a standard PC within a time of no longer than 0.1 s. The axle detection algorithm, supplemented with the algorithm for measuring the distance between axles, can be used in an automatic vehicle classification system.

## 5. Conclusions

A new method for measuring impedance profiles of an IL sensor has been presented, and an electronic circuit for this method has been proposed. Simulations carried out using a simplified model of the system confirmed that it performed correctly. The multichannel system constructed was designed to work with a narrow IL sensor. The system successfully fulfils its task, demonstrating the stability and repeatability of results. This paper presents an algorithm for detecting the number of vehicle axles using profiles split into impedance components: R-profile and X-profile. As was shown, the novel algorithm is characterized by a very high detection accuracy of no less than 98.8% with reference to the real number of vehicle axles, and of 71.8% for vehicles travelling with a lifted axle. The research on axle detection accuracy was carried out on 4000 vehicles travelling in real traffic conditions on an extra-urban road with a speed limit of 70 km/h. In several vehicle groups (4-axle and 5-axle trucks), the axle detection accuracy reached 100%.

## Figures and Tables

**Table 1:** Vehicle groups in the test base, where *N*—number of vehicles in the group.

No.	Vehicle Description in Group	*N*
1	2-axle passenger vehicle with aluminum rims	1000
2	2-axle passenger vehicle with steel rims	1228
3	2-axle delivery van	630
4	2-axle truck and 2-axle bus	175
5	3-axle truck and 3-axle bus	316
6	5-axle truck with lifted axle (4 axles)	234
7	5-axle truck	406
8	4-axle truck	11

**Table 2:** Optimal values of algorithm-core setting parameters, determined for maximum accuracy (10) of axle detection in groups considered.

No.	Vehicle Description in Group	Level	Hist	Gain	Suspension
1	2-axle passenger vehicle with aluminum rims	1.8	0.66	0.50	Low
2	2-axle passenger vehicle with steel rims	1.9	0.73	0.51	
3	2-axle delivery van	1.8	0.54	0.54	
4	2-axle truck and 2-axle bus	0.8	0.41	0.18	High
5	3-axle truck and 3-axle bus	0.8	0.23	0.24	
6	5-axle truck with lifted axle (4 axles)	0.4	0.02	0.68	
7	5-axle truck	0.8	0.34	0.27	

**Table 3:** Vehicle axle detection accuracy (10) using an algorithm-core verified for 4000 vehicles.

No.	Vehicle Description in Group	Number of Axles Detected					
		1	2	3	4	5	
1	2-axle passenger vehicle with aluminum rims	3.8	**87.0**	7.0	1.9	0.3	Accuracy (10) in [%]
2	2-axle passenger vehicle with steel rims	2.9	**89.3**	6.3	1.1	0.0	
3	2-axle delivery van	0.8	**97.3**	1.7	0.2	0.0	
4	2-axle truck and 2-axle bus	3.4	**95.4**	0.6	0.6	0.0	
5	3-axle truck and 3-axle bus	0.0	0.3	**99.1**	0.6	0.0	
6	5-axle truck with lifted axle (4 axles)	0.0	0.0	0.4	97.4	**2.2**	
7	5-axle truck	0.0	0.0	0.0	0.0	**100.0**	
8	4-axle truck	0.0	0.0	0.0	**100.0**	0.0	

**Table 4:** Vehicle axle detection accuracy (10) for a set of data from 4000 vehicles, using the algorithm from Figure 8.

No.	Vehicle Description in Group	Number of Axles Detected					
		1	2	3	4	5	
1	2-axle passenger vehicle with aluminum rims	0.0	**99.0**	1.0	0.0	0.0	Accuracy (10) in [%]
2	2-axle passenger vehicle with steel rims	0.0	**99.3**	0.7	0.0	0.0	
3	2-axle delivery van	0.0	**99.7**	0.3	0.0	0.0	
4	2-axle truck and 2-axle bus	0.0	**98.8**	0.6	0.6	0.0	
5	3-axle truck and 3-axle bus	0.0	0.3	**99.1**	0.6	0.0	
6	5-axle truck with lifted axle (4 axles)	0.0	0.0	0.4	**27.8**	**71.8**	
7	5-axle truck	0.0	0.0	0.0	0.0	**100.0**	
8	4-axle truck	0.0	0.0	0.0	**100.0**	0.0	

## References

[1] Klein L.A. (2001). Sensor Technologies and Data Requirements for ITS.

[2] Coifman B. (2001). Improved Velocity Estimation Using Single Loop Detectors. Transp. Res. Part A.

[3] Coifman B., Dhoorjaty S., Lee Z. (2003). Estimating Median Velocity Instead of Mean Velocity at Single Loop Detectors. Transp. Res. Part C.

[4] Hoeschen B., Erker M., Janson B., Medland R. (2005). Best Practices Guidebook: Collecting Short Duration Manual Vehicle Classifications Counts on High Volume Urban Facilities.

[5] Burnos P. (2010). Alternative Automatic Vehicle Classification Method. Metrol. Meas. Syst..

[6] Yang S., Kalpakis K., Biem A. (2014). Detecting Road Traffic Events by Coupling Multiple Timeseries with a Nonparametric Bayesian Method. IEEE Trans. Intell. Transp. Syst..

[7] Jeng S.-T., Chu L. (2015). Tracking Heavy Vehicles Based on Weigh-In-Motion and Inductive Loop Signature Technologies. Trans. Intell. Transp. Syst..

[8] Hernandez S.V., Tok A., Ritchie S.G. (2016). Integration of Weigh-in-Motion (WIM) and inductive signature data for truck body classification. Transp. Res. Part C.

[9] Burnos P., Gajda J., Marszałek Z., Piwowar P., Sroka R., Stencel M., Zeglen T. (2011). Road Traffic Parameters Measuring System with Variable Structure. Metrol. Meas. Syst..

[10] Nishibe Y., Ohta N., Tsukada K., Yamadera H., Nonomura Y., Mohri K., Uchiyama T. (2004). Sensing of Passing Vehicles Using a Lane Marker on Road with a Built-in Thin Film MI Sensor and Power Source. IEEE Trans. Veh. Technol..

[11] Nishibe Y., Yamadera H., Ohta N., Tsukada K., Ohmura Y. (2003). Magneto-impedance effect of a layered CoNbZr amorphous film formed on a polyimide substrate. IEEE Trans. Magn..

[12] Atkinson D., Squire P.T., Maylin M.G., Goreb J. (2000). An integrating magnetic sensor based on the giant magneto-impedance effect. Sens. Actuators A Phys..

[13] Kurlyandskaya G.V., de Cos D., Volchkov S.O. (2009). Magnetosensitive transducers for nondestructive testing operating on the basis of the giant megnetoimpedance effect: A review. Russ. J. Non-Destruct. Test..

[14] Jogschies L., Klaas D., Kruppe R., Rittinger J., Taptimthong P., Wienecke A., Rissing L., Wurz M.C. (2015). Recent Developments of Magnetoresistive Sensors for Industrial Applications. Sensors.

[15] Rifai D., Abdalla A.N., Ali K., Razali R. (2016). Giant Magnetoresistance Sensors: A Review on Structures and Non-Destructive Eddy Current Testing Applications. Sensors.

[16] Lu C.C., Huang J., Chiu P.K., Chiu S.L., Jeng J.T. (2014). High-Sensitivity Low-Noise Miniature Fluxgate Magnetometers Using a Flip Chip Conceptual Design. Sensors.

[17] Gajda J., Piwowar P., Sroka R., Stencel M., Zeglen T. (2012). Application of Inductive Loops as Wheel Detectors. Transp. Res. Part C.

[18] Oh C., Ritchie S.G., Jeng S.-T. (2007). Anonymous Vehicle Reidentification Using Heterogeneous Detection Systems. IEEE Trans. Intell. Transp. Syst..

[19] Oh C., Ritchie S.G. (2007). Recognizing Vehicle Classification Information from Blade Sensor Signature. Pattern Recognit. Lett..

[20] Marszalek Z., Sroka R. Signal Fusion of Changes in the Inductive Loop Impedance Components for Vehicle Axle Detection. Proceedings of the IEEE 21st International Conference on Methods and Models in Automation and Robotics.

[21] Stanczyk D. (1997). Device to Detect Particularly One or Several Wheels of a Vehicle or of a Wheeled Mobile Engine and Process for Using This Device. US Patent.

[22] Lees R.H. (2002). Inductive Loop Sensor for Traffic Detection, and Traffic Monitoring Apparatus and Method Using Such a Loop Sensor. US Patent.

[23] Lees R.H., Lees R.A. (2002). Loop Sensing Apparatus for Traffic Detection. US Patent.

[24] Peek SmartToll (2010). Advanced Axle-Detection Tolling Classifier, Loop Installation Manual.

[25] Lamas-Seco J.J., Castro P.M., Dapena A., Vazquez-Araujo F.J. (2017). Multi-loop inductive sensor model for vehicle traffic applications. Sens. Actuators A Phys..

[26] Anderson R.L. (1970). Electromagnetic Loop Vehicle Detectors. IEEE Trans. Veh. Technol..

[27] Day C., Brennan T., Harding M., Premachandra H., Jacobs A., Bullock D., Krogmeier J., Sturdevant J. (2009). Three-Dimensional Mapping of Inductive Loop Detector Sensitivity with Field Measurement. Transp. Res. Rec..

[28] Marszalek Z. (2018). Maxwell-Wien bridge with vector voltmeter system for measurement small and rapid changes in inductive-loop sensor impedance components. Measurement.

